# Knowledge, attitude, and practices of parents regarding the red flags of developmental milestones in children aged 0–5 years in Karachi, Pakistan: a cross-sectional study

**DOI:** 10.1186/s12887-024-04574-9

**Published:** 2024-02-14

**Authors:** Raman Kumar, Malaika Ali, Muhammad Saad Pasha, Haya Waseem Ansari, Noureen Durrani

**Affiliations:** Liaquat National Hospital and Medical College, Karachi, Pakistan

**Keywords:** Developmental milestones, Parents, Red flags, Knowledge, Attitude, Practices, Developmental delays

## Abstract

**Background:**

Developmental delays in children are assessed in four basic domains: gross motor, fine motor, social, and language. Early years of life are crucial in a child’s development, so it is imperative that parents be aware of developmental milestones to facilitate early diagnosis and treatment in case of a developmental delay. This study assessed parental knowledge, attitude, and practices regarding children’s developmental milestones and associated “red flags”.

**Methods:**

A cross-sectional study was conducted at the Department of Pediatrics at Liaquat National Hospital, Karachi. 390 parents, who had at least one child under 5 years of age, with no diagnosed developmental delay, were interviewed during outpatient clinic visits. The questionnaire consisted of three components to assess parental knowledge, attitude, and practices.

**Results:**

59% and 54% of parents had poor knowledge of gross and fine motor milestones respectively; In the social domain, 56% of the respondents had inadequate knowledge. 42% had inadequate knowledge of language milestones; 29% of parents strongly agreed that their pediatricians provide satisfactory information regarding red flags of developmental milestones. 60% of parents strongly agreed that their child’s developmental delay would be a cause of concern for them. In the case of developmental delay, 55% of parents said they would consult a general pediatrician, 11% preferred a pediatric neurologist, 21% opted for a developmental pediatrician and 13% opted for a family physician. Residence and family systems were found to be associated with language-related milestones with significantly higher odds of knowledge among urban residents than rural ones and a significantly lower likelihood of language milestones knowledge among joint families than nuclear families. Female gender was found to be significantly associated with positive attitude.

**Conclusion:**

The majority of our respondents showed considerably poor knowledge regarding developmental milestones. This highlights the need to devise ways to educate parents on this subject to enable them to vigilantly monitor their child’s developmental status and any associated abnormalities and ultimately facilitate the right course of action.

**Supplementary Information:**

The online version contains supplementary material available at 10.1186/s12887-024-04574-9.

## Background

Children are screened for developmental delays in 4 basic domains, gross motor, and fine motor, social and verbal. Once the delays are identified, further detailed evaluation is carried out in cognition, language, motor skills, and social and emotional behavioral domains. The term “development” is defined as the progress of a child in all areas of human functioning [1]. Early years of life play a crucial role in the mental and physical development of a child and the two most important pillars in the proper development of children are parents and doctors [2].

Generally, parents are the ones who spend most of the time with their children and are involved in raising them so they should have the best knowledge related to developmental signs as parental concern about a child’s development can be used as a reliable predictor of actual developmental delays [3]. There are three channels for red flags in developmental milestones to get noticed, either via parents, physicians during routine check-ups, or if teachers notice [4].

Parents must recognize the need to take their child for routine check-ups to a physician and immediately, in case they notice a red flag. One study from 2017 showed that without routine screening, only 29% of children with developmental issues were identified before kindergarten [5]. This allows us to assume that a large number of children with developmental issues were not identified before kindergarten due to parents being unable to identify the red flags. Although the major responsibility of recognizing developmental milestones and any delay in them is the responsibility of the healthcare system, cases, where parents fail to get their children routinely monitored for growth and development by healthcare professionals and not being able to recognize major red flags of development by themselves, can lead to serious consequences in a child’s health.

Studies report that early recognition of red flags in the social, motor, and cognitive aspects of development helps in better and proactive management and therapies of children for example autism. There is, therefore, a need for early intervention. Early intervention and treatment can be very beneficial for the child and the family, according to research done in the USA in 2015, about 3.5% to 15% of children have developmental disabilities who need early intervention but only 2.8% of these toddlers and infants receive early interventional services [6].

Meanwhile, there is a lack of validated and reliable measurement tools to monitor the developmental progress of the younger age groups that can be easily adapted to regions that may be culturally, socially, and economically very diverse from the Western world. Assessment tools that were designed for resource-rich countries cannot really be used to make generalizations elsewhere. This poses a great challenge to the enforcement of programs to support childhood development which can be the reason why in low- and middle-income countries, such as Pakistan, over 250 million children are at risk of not reaching their developmental potential by 5 years of age. Moreover, the first alarm that children are not developing as they should is noticed usually when they join school which is already too late for intervention [7]. Hence, parents’ early recognition of developmental delays may result in earlier diagnosis and early intervention. With an ever-increasing population and a high birth rate (3.51 births per woman), it is the need of the hour in Pakistan, to know how to improve the conditions for children in which they are brought up so that they can become healthy and socially productive members of society. According to UNICEF's Early Childhood Development Index (ECDI), reports of nearly 100,000 caregivers show that 36.8% of children aged 3 and 4 years in LMICs do not achieve basic cognitive and socio-emotional skills. ECDI scores are positively associated with caregiver–child joint activities such as reading and playing which simply highlights the integral role that a positive relationship between the child and caregiver has on the child’s social and cognitive development [8]. It is incumbent upon all parents to know what they should expect their child to be able to do by a certain age and what they can do to help. They must also be well informed about when and who they should inform in case of delay. Parents noticing red flags at the right time and allowing for early intervention can be the turning point in deciding the prognosis of children and supporting them in becoming independent members of society and preventing disability [9]. This study aims to assess the baseline knowledge of parents about the red flags in developmental milestones in childhood and to learn about parents’ developmental knowledge-seeking attitudes and healthcare usage regarding developmental care [10].

## Material and methods

### Study design, duration, and setting

This is a cross-sectional study conducted in the pediatric department of a private, tertiary care hospital namely Liaquat National Hospital, Karachi from September 2021 to February 2022. Liaquat National Hospital comprises 700 beds in total with nearly 5,000 total workforce. The total number of beds operational in the pediatric unit is 77 with 13 pediatricians and 35 nurses. The questionnaire was handed to the parents who had children under the age of five and who visited the pediatric outpatient department. This questionnaire, which was developed by the authors, included a consent form in the beginning followed by a set of questions regarding developmental milestones.

### Study population

Parents who had at least one child under the age of five were included. This was to minimize recall bias as we assumed that parents who have all children above the age of five years may not clearly remember the age at which developmental milestones are achieved. Parents who have children with a developmental delay that was formally, clinically diagnosed by a healthcare practitioner, were excluded as they are already aware of delayed milestones. The data collection procedure lasted from September 2021 till February 2022.

### Survey questionnaire

A structured self-administered questionnaire was designed by conducting a detailed literature search related to red flag signs for developmental delay in children published by the Royal Australian College of General Practitioners [11]. For each of the four developmental domains, questions pertaining to red flags of developmental delays were framed in coherence with The Royal Australian College of General Practitioners [11] and questions regarding parental knowledge were written as multiple-choice questions in accordance with the developmental milestones mentioned in the Current Diagnosis & treatment pediatrics, Kliegman RM. Nelson Textbook of Pediatrics and Khan PA. Basis of Pediatrics [12–14]. Online available booklet related to guidelines for preventive activities. The content of the questionnaire was validated by the field experts who were not part of this study. The questionnaire comprised four sections which included demographics, knowledge, parental practices, and parental attitude regarding developmental milestones. There was a total of 68 questions. The demographic part included a total of 15 questions that inquired about gender, age, residence, number of children, education, language, and family system. The knowledge section was further divided into four sections including gross motor, fine motor, social, and language milestones, and each of these sections contained a total of 11 questions. Each question in the knowledge section was a multiple choice where the participant was asked a developmental milestone regarding each domain and 4 options were given from which they had to choose. A score of one was assigned to each correct answer. Knowledge was labeled as adequate for each knowledge component when the score was found to be at least 80% of the total score i.e., ≥ 9. Otherwise, knowledge was considered inadequate. Attitude towards red flags of developmental milestones was assessed with 5 questions and the answer options included strongly agree, agree, neutral, disagree, and strongly disagree. A score of one was assigned to each correct answer. The attitude was labeled as positive for each attitude component when the score was found to be at least 80% of the total score i.e., ≥ 4. Otherwise, the attitude was considered negative. Parental practices had 5 questions as well however the answer option was either multiple choice or agree and disagree. A score of one was assigned to each correct answer. The practice was labeled as satisfactory for each practice component. When the score was found to be at least 80% of the total score i.e., ≥ 4. Otherwise, the practice was considered unsatisfactory. The reliability of the questionnaire was checked through Cronbach’s alpha value during the pilot study on 30 participants and the calculated Cronbach alpha value was 0.7. Cronbach alpha values for subsections of knowledge including gross motor, fine motor, social, and language milestones are 0.74, 0.75, 0.79, and 0.76 respectively. Cronbach alpha for attitude and practice was 0.82 and 0.78 respectively.

### Sample size calculation

Since no similar study has been conducted in Pakistan, we assumed that 50% of people would have the adequate knowledge, positive attitudes and satisfactory practices regarding red flags of child development. Using a 95% confidence interval and a precision of 5%, a sample of 384 patients was required. Sample size estimation was performed on the online available calculator Open-Epi.

### Data collection

The data was collected by authors MA, MSP and HWA, currently medical students in their final year. No other healthcare staff were involved in the data collection or participant recruitment. Participants were handed out forms which they filled themselves if literate and if they could not read, they were interviewed. The questionnaire began with the sub-section on informed consent and each participant had to sign the form under it. In the outpatient department, it was convenience sampling, and the participants had a separate room. Not all participants were able to complete the written survey in English which we had anticipated and therefore had an Urdu translation ready. Each questionnaire took approximately 15 -20 min to complete. Parents were excluded by being asked questions relevant to the exclusion criteria. The data was kept in a password-protected computer which was only accessible to the principal investigator. Moreover, the medical record number was also tagged with other serial numbers to conceal the identity of patients.

### Data management and analysis

Data was analyzed using IBM SPSS version 21 (Armonk, New York). Frequencies and percentages were computed for categorical variables. Numerical variables were presented as mean ± standard deviation or median with interquartile range as appropriate. Participants’ characteristics were compared among two knowledge groups using chi-square of Fisher exact test. A *p*-value less than or equal to 0.05 was taken as statistically significant.

## Results

A total of 390 participants responded to a survey with a 100% completion rate. The median age of study participants was 33 (IQR = 29 – 37) years whereas the age range was 20–59 years. One male was 59 years old and reported that his youngest child was four years old. Education levels of participants were variable throughout the sample including illiterate (4.6%), primary education (9.7%), secondary education (29.5%), graduation (38.5%), and post-graduates (17.7%). Most of the responses were received from mothers (55.4%) and urban areas (92.6%). Participants of multiple ethnicities responded to the survey including Urdu speaking (61.8%), Punjabi (19.7%), Sindhi (8.2%), Pashto (5.9%), Memon (2.1%), Balochi (1.5%), Hindko (0.5) and Kashmiri (0.3). Most of them were Muslims (96.7) whereas few were Hindu (2.6%), Christians (0.5), and Parsi (0.3%). One-third (33.6%) of the participants were first-time parents, and 84.1% of our participants reported that they did not have a consanguineous marriage and were living in a nuclear family system (69%). The median number of children per respondent was 2(IQR = 1–3) and the median age of children was also 2 (IQR = 1–3).The primary caretaker of children was the mother (83.3%), grandmother (6.9%), babysitter or daycare (6.7%), father (1.3%), aunt (1%), elder sibling (0.5%) and grandfather (0.3%).

### Knowledge related to different parameters of developmental delay.

**Fig. 1:**
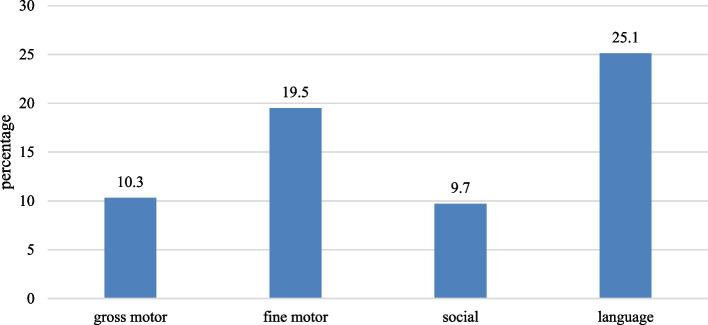
Frequency of overall knowledge for different developmental domains

The median knowledge score for gross motor, fine motor, and social milestones was 4(IQR- 3–5) while the median score for language was 5 (IQR 4–6). Table 1 depicts the knowledge on different items of developmental delay. Regarding knowledge of gross motor milestones, more than half had knowledge of the correct age a child should be able to lift his head and chest while lying on his stomach (64.1%), should be able to walk with support e.g., with one hand-held or by holding onto furniture (70%). On the domain of knowledge related to fine motor milestones, more than half had correct knowledge of the age a child should be able to reach for objects (57.9%) and the age a child should be able to use scissors to cut out a figure/picture (56.7%). On the component of social milestones, the majority had knowledge of the correct age a child should be toilet trained (65.1%) and should be able to dress and undress except by tying shoelaces (71.5%). On the domain of language milestone, most had correct knowledge of the age does a child say his first word (67.9%), the age at which a child should have a vocabulary of 50 or more words (83.3%), age a child enjoys being read to (80.5%), age a child should be able to give his/her full name and age (57.2%), age a child should be able to call his mother and father “mama” and “dada” respectively (61.8%) and age a child should be able to respond to simple instructions like “sit down” or “bring it here” (58.5%) Fig. 1.

**Table 1:** Knowledge of different items of developmental delay

Knowledge Items	Correct n(%)	Incorrect n(%)
**Knowledge regarding gross motor milestones**		
By what age should a child be able to lift his head and chest while lying on his stomach?	250(64.1)	140(35.9)
By what age should a child be able to sit without support?	74(19)	316(81)
By what age should a child be able to pedal a tricycle?	182(46.7)	208(53.3)
By what age should a child be able to walk along a straight line?	46(11.8)	344(88.2)
By what age should a child be able to walk with support e.g. with one hand-held or by holding onto furniture?	273(70)	117(30)
By what age should a child be able to roll over in either direction?	146(37.4)	244(62.6)
By what age should a child be able to walk up and down stairs with one hand-held?	158(40.5)	232(59.5)
By what age should a child walk independently?	144(36.9)	246(63.1)
By what age should a child be able to stand on one foot for a few seconds?	182(46.7)	208(53.3)
**Knowledge regarding fine motor milestones**		
By what age should a child be able to reach for objects e.g. rattle after seeing them?	226(57.9)	164(42.1)
By what age should a child be able to do a pincer grasp i.e. hold an object with index finger and thumb?	169(43.3)	221(56.7)
By what age should a child be able to copy a circle?	179(45.9)	211(54.1)
By what age should a child be able to scribble with a pen/pencil?	168(43.1)	222(56.9)
By what age should a child put objects in his/her mouth?	139(35.6)	251(64.4)
By what age should a child be able to transfer objects from one hand to the other?	196(50.3)	194(49.7)
By what age should a child be able to copy a square or a triangle?	167(42.8)	223(57.2)
By what age should a child be able to use scissors to cut out a figure/picture?	221(56.7)	169(43.3)
By what age should a child be able to close a box with a lid??	154(39.5)	236(60.5)
**Knowledge regarding social and help milestones**		
By what age should a child be able to participate in group play?	53(13.6)	337(86.4)
By what age should a child be able to drink from a cup and use a spoon?	194(49.7)	196(50.3)
By what age should a child be able to smile spontaneously?	181(46.4)	209(53.6)
By what age does a child develop a fear of strangers?	179(45.9)	211(54.1)
By what age should a child be able to point to a desired object?	138(35.4)	252(64.6)
By what age should a child be toilet trained?	254(65.1)	136(34.9)
By what age should a child be able to remove garments like shoes and socks?	109(27.9)	281(72.1)
By what age does a child normally begin to recognize his/her caregiver?	132(33.8)	258(66.2)
By what age should a child be able to dress and undress except tying shoe laces?	279(71.5)	111(28.5)
**Knowledge regarding language milestones**		
By what age does a child begin to respond to his/her own name?	65(16.7)	325(83.3)
By what age does a child say his first word?	265(67.9)	125(32.1)
By what age should a child be able to vocalize (i.e. make audible response/sounds) when talked to?	179(45.9)	211(54.1)
By what age should a child have a vocabulary of 50 or more words?	325(83.3)	65(16.7)
By what age should a child be able to tell which hand is right and which is left?	194(49.7)	196(50.3)
By what age does a child enjoy being read to?	314(80.5)	76(19.5)
By what age should a child be able to give his/her full name and age?	223(57.2)	167(42.8)
By what age should a child be able to call his mother and father “mama” and “dada” respectively?	241(61.8)	149(38.2)
By what age should a child be able to respond to simple instructions like “sit down” or “bring it here”?	228(58.5)	162(41.5)

### Association of participants’ features with knowledge of gross motor, fine motor, social and language milestones

None of the participant features was found to be associated with knowledge of gross motor (Table 2) and fine motor (Table 3). None of the participants’ features was associated with knowledge of social milestones (Table 4). Residence and family systems were found to be associated with language-related milestones with significantly higher odds of knowledge among urban residents than rural ones and a significantly lower likelihood of language milestones knowledge among joint families than nuclear families (Table 5).

**Table 2:** Association of participants’ features with knowledge regarding gross motor

Variables	Groups	Knowledge status		OR (95% CI)	*p*-value
		**Adequate n(%)**	**Non-adequate n(%)**		
Age (in years)#	-	32 (28.3—36)	33 (29—37)	1 (0.95—1.05)	0.942
Total children#	-	2 (1 -2)	2 (1—3)	0.82 (0.60—1.12)	0.206
Gender	Male	16(9.2)	158(90.8)	0.81 (0.42—1.58)	0.536
	Female	24(11.1)	192(88.9)	Ref	
Residence	Urban	40(11.1)	321(88.9)	-	-
	Rural	0(0)	29(100)	-	-
Language	Urdu	25(10.4)	216(89.6)	1.85 (0.24—14.56)	0.558
	Punjabi	12(15.6)	65(84.4)	2.95 (0.36—24.41)	0.315
	Sindhi	1(3.1)	31(96.9)	0.52 (0.03—12.52)	0.648
	Pashto	1(4.3)	22(95.7)	0.73 (0.04—12.52)	0.826
	Others	1(5.9)	16(94.1)	Ref	
Education	Illiterate	1(5.6)	17(94.4)	0.35 (0.04—2.91)	0.329
	Primary	1(2.6)	37(97.4)	0.16 (0.02—1.30)	0.086
	Secondary	8(7)	107(93)	0.44 (0.17—1.18)	0.103
	Graduate	20(13.3)	130(86.7)	0.91 (0.40—2.06)	0.817
	Postgraduate	10(14.5)	59(85.5)	Ref	
Family system	Joint	17(14)	104(86)	1.75 (0.90—3.41)	0.101
	Nuclear	23(8.6)	246(91.4)	Ref	

*CI* Confidence interval, *OR* Odds ratio, *Ref* Reference category, *#* Non-normal data presented as median (inter-quartile range)

**Table 3:** Association of participant features with knowledge of fine motor milestone

Variables	Groups	Knowledge status		OR (95% CI)	*p*-value
		**Adequate n(%)**	**Non-adequate n(%)**		
Age (in years)#	-	33 (28.3—37)	33 (29—37)	1.01 (0.97—1.05)	0.800
Total children#	-	2 (1—3)	2 (1—3)	0.97 (0.78—1.21)	0.799
Gender	Male	32(18.4)	142(81.6)	0.88 (0.53—1.46)	0.624
	Female	44(20.4)	172(79.6)		
Residence	Urban	74(20.5)	287(79.5)	3.48 (0.81—14.97)	0.094
	Rural	2(6.9)	27(93.1)	Ref	
Language	Urdu	50(20.7)	191(79.3)	1.22 (0.34—4.42)	0.760
	Punjabi	17(22.1)	60(77.9)	1.32 (0.34—5.14)	0.687
	Sindhi	5(15.6)	27(84.4)	0.86 (0.18—4.12)	0.855
	Pashto	1(4.3)	22(95.7)	0.21 (0.02—2.25)	0.198
	Others	3(17.6)	14(82.4)	Ref	
Education	Illiterate	3(16.7)	15(83.3)	0.72 (0.18—2.82)	0.637
	Primary	7(18.4)	31(81.6)	0.81 (0.30—2.21)	0.685
	Secondary	20(17.4)	95(82.6)	0.76 (0.36—1.60)	0.468
	Graduate	31(20.7)	119(79.3)	0.94 (.47—1.88)	0.856
	Postgraduate	15(21.7)	54(78.3)	Ref	
Family system	Joint	19(15.7)	102(84.3)	0.69 (0.39—1.27)	0.207
	Nuclear	57(21.2)	212(78.8)	Ref	

*CI* Confidence interval, *OR* Odd ratio, *Ref* Reference category, *#* Variables are expressed as median with inter-quartile range

**Table 4:** Association of participant features with knowledge of social milestones

Variables	Groups	Knowledge status		OR (95% CI)	*p*-value
		**Adequate n(%)**	**Non-adequate n(%)**		
Age (in years)#	-	31 (28—37)	33 (29—37)	0.99 (0.94—1.05)	0.864
Total children#	-	2 (1—3)	2 (1—3)	0.87 (0.63—1.18)	0.359
Gender	Male	16(9.2)	158(90.8)	0.89 (0.45—1.76)	0.743
	Female	22(10.2)	194(89.8)	Ref	
Residence	Urban	36(10)	325(90)	1.50 (0.34—6.55)	0.593
	Rural	2(6.9)	27(93.1)	Ref	
Language	Urdu	24(10)	217(90)	1.77 (0.23—13.94)	0.588
	Punjabi	9(11.7)	68(88.3)	2.12 (0.25—17.94)	0.491
	Sindhi	2(6.3)	30(93.8)	1.07 (0.09—12.69)	0.959
	Pashto	2(8.7)	21(91.3)	1.52 (0.13—18.32)	0.740
	Others	1(5.9)	16(94.1)	Ref	
Education	Illiterate	1(5.6)	17(94.4)	0.62 (0.07—5.48)	0.665
	Primary	1(2.6)	37(97.4)	0.28 (0.03—2.45)	0.252
	Secondary	13(11.3)	102(88.7)	1.33 (0.48—3.70)	0.574
	Graduate	17(11.3)	133(88.7)	1.34 (0.51—3.57)	0.555
	Postgraduate	6(8.7)	63(91.3)	Ref	
Family system	Joint	16(13.2)	105(86.8)	1.71 (0.86—3.34)	0.123
	Nuclear	22(8.2)	247(91.8)	Ref	

*CI* Confidence interval, *OR* Odd ratio, *Ref* Reference category, *#* Variables are expressed as median with inter-quartile range

**Table 5:** Association of participant features with knowledge of language milestone

Variables	Groups	Knowledge status		OR (95% CI)	*p*-value
		**Adequate n(%)**	**Non-adequate n(%)**		
Age (in years)#	-	34 (28—39)	33 (29—37)	1.03 (0.99—1.06)	0.156
Total children#	-	2 (1—3)	2 (1—3)	0.88 (1.07—1.31)	0.482
Gender	Male	46(26.4)	128(73.6)	1.13 (0.72—1.79)	0.593
	Female	52(24.1)	164(75.9)	Ref	
Residence	Urban	97(26.9)	264(73.1)	10.29 (1.38—76.64)	*0.023
	Rural	1(3.4)	28(96.6)	Ref	
Language	Urdu	62(25.7)	179(74.3)	2.60 (0.58—11.68)	0.213
	Punjabi	26(33.8)	51(66.2)	3.82 (0.81—18)	0.090
	Sindhi	7(21.9)	25(78.1)	2.10 (0.39—11.46)	0.391
	Pashto	1(4.3)	22(95.7)	0.34 (0.03—4.11)	0.397
	Others	2(11.8)	15(88.2)	Ref	
Education	Illiterate	4(22.2)	14(77.8)	1.03 (0.30—3.59)	0.965
	Primary	9(23.7)	29(76.3)	1.12 (0.44—2.87)	0.817
	Secondary	26(22.6)	89(77.4)	1.05 (0.51—2.16)	0.891
	Graduate	44(29.3)	106(70.7)	1.49 (0.76—2.92)	0.241
	Postgraduate	15(21.7)	54(78.3)	Ref	
Family system	Joint	22(18.2)	99(81.8)	0.56 (0.33—0.96)	*0.035
	Nuclear	76(28.3)	193(71.7)	Ref	

*CI* Confidence interval, *OR* Odd ratio, *Ref* Reference category, # Variables are expressed as median with inter-quartile range

Table 6 displays the response of study participants regarding their attitude towards developmental delay. 64.4% of participants said that they had looked up information for children's’ developmental milestones themselves while 73.4% of the parents agreed that Pediatricians have provided them with satisfactory and sufficient information regarding children's’ developmental milestones and their red flags. Only 55.9% of the parents agreed that in case of a positive family history of developmental delay, they will get their child developmental delay assessment. 42.6% of the parents agreed that delays in motor development can be a strong indication of physical disability while 36.4% of the parents were neutral about it meaning they were not sure of the answer. 31% of the participants agreed that social and verbal development delays can lead to the child becoming deaf and/or mute while 32.1% of the participants disagreed. Table 7 shows the association of participants’ features with positive attitudes. On the multivariable model, the male gender was found to be significantly associated with positive attitudes regarding supporting children’s development.

**Table 6:** Response distribution of study participants for attitude items

Attitude items	Frequency (%)
**Have you ever looked up/sought information for children's’ developmental milestones yourself**	
Yes	251(64.4)
No	139(35.6)
**Pediatricians (children's doctors) provides satisfactory and sufficient information regarding children's’ developmental milestones and their red flags**	
Strongly agree	111(28.5)
Agree	175(44.9)
Neutral	77(19.7)
Disagree	13(3.3)
Strongly disagree	14(3.6)
**In case of positive family history, will you get the DD assessment done?**	
Strongly agree	84(21.5)
Agree	134(34.4)
Neutral	148(37.9)
Disagree	16(4.1)
Strongly disagree	8(2.1)
**You consider delays in motor development to be a strong indication of physical disability**	
Strongly agree	64(16.4)
Agree	102(26.2)
Neutral	142(36.4)
Disagree	62(15.9)
Strongly disagree	20(5.1)
**You consider that social and verbal development delays can lead to the child becoming deaf and/or mute**	
Strongly agree	44(11.3)
Agree	77(19.7)
Neutral	144(36.9)
Disagree	74(19)
Strongly disagree	51(13.1)

*CI* Confidence interval, *OR* Odd ratio, *Ref* Reference category, # Variables are expressed as median with inter-quartile range

**Table 7:** Association of participants’ features with adequate attitude

Variables	Attitude status		OR (95% CI)	*p*-value	aOR (95% CI)	*p*-value
	**Positive n(%)**	**Negative n(%)**				
Age^#^	33 (28—37)	33 (29—37)	1 (0.97—1.04)	0.974	-	-
Number of children^#^	2 (1—3)	2 (1—3)	0.90 (0.74—1.09)	0.266	-	-
**Gender**						
male	40(23)	134(77)	0.57 (0.37—0.90)	*0.016	0.62 (0.39—0.98)	*0.041
female	74(34.3)	142(65.7)	Ref		Ref	
**Residence**						
urban	102(28.3)	259(71.7)	0.56 (0.26—1.21)	0.139	0.71 (0.32—1.59)	0.405
rural	12(41.4)	17(58.6)	Ref		Ref	
**Languages**						
Urdu	67(27.8)	174(72.2)	0.92 (0.31—2.72)	0.886	-	-
Punjabi	29(37.7)	48(62.3)	1.45 (0.46—4.54)	0.523	-	-
Sindhi	8(25)	24(75)	0.80 (0.22—2.98)	0.739	-	-
Pashto	5(21.7)	18(78.3)	0.67 (0.16—2.81)	0.581	-	-
Others	5(29.4)	12(70.6)	Ref		Ref	
**Education**						
Illiterate	4(22.2)	14(77.8)	0.58 (0.17—1.93)	0.368	-	-
Primary	14(36.8)	24(63.2)	1.17 (0.51—2.67)	0.715	-	-
Secondary	27(23.5)	88(76.5)	0.61 (0.32—1.19)	0.147	-	-
Graduate	46(30.7)	104(69.3)		0.693	-	-
Postgraduate	23(33.3)	46(66.7)	Ref		Ref	
**Family system**						
Joint	43(35.5)	78(64.5)	1.54 (0.97—2.44)	0.067	1.36 (0.84—2.20)	0.207
Nuclear	71(26.4)	198(73.6)	Ref		Ref	

*CI* Confidence interval, *OR* Odd ratio, *Ref* Reference category, *#* Variables are expressed as median with inter-quartile range

### The practice of study participants’ developmental delay in children and association of socio-demographic features with satisfactory practices

Table 8 displays the response of study participants for practice toward developmental delay. 51.5% of the parents said they visited pediatricians about 1–2 times a year while 37.4% parents consult them more than 2 times a year. Only 5.1% parents said they visit as per need. The percentage of parents who spent more than 8 h in a day with their child was 34.6% while those who spent around 4–8 h was 25.6%. 84.7% of the parents agreed that they will get a developmental delay assessment done in case they notice a delay in any of the domains. 90.8% of the parents agreed that spending time interacting with their child improves their language and social development and in case of a developmental delay 55.1% of the parents will first consult a general pediatrician. Table 9 shows the association of participants’ features with adequate practice status. On the multivariable model, female gender and education was found to be significantly associated with satisfactory practice.

**Table 8:** Response distribution of study participants for practice items

Practice items	Frequency (%)
**How many times do you visit/consult a pediatrician (children's doctor) in a year on average?**	
None	23(5.9)
1–2 times	201(51.5)
> 2 times	146(37.4)
as per need	20(5.1)
**How much time do you spend with your child daily**	
< 1 h	5(1.3)
1–2 h	41(10.5)
2–4 h	109(27.9)
4–8 h	100(25.6)
> 8 h	135(34.6)
**On identifying delay in any domain, will you get the DD assessment done?**	
Strongly agree	235(60.3)
Agree	95(24.4)
Neutral	49(12.6)
Disagree	7(1.8)
Strongly disagree	4(1)
**Do you spend time interacting with the child with an intent to improve their language and social development?**	
Strongly agree	191(49)
Agree	163(41.8)
Neutral	34(8.7)
Disagree	1(0.3)
Strongly disagree	1(0.3)
**Who should one consult if their child has a developmental delay?**	
General paediatrician	215(55.1)
Paediatric neurologist	44(11.3)
Developmental paediatrician	82(21)
Family physician	49(12.6)

**Table 9:** Association of participants’ features with adequate practice status

Variables	Practice status		OR (95% CI)	*p*-value	aOR (95% CI)	*p*-value
	**Satisfactory n(%)**	**Unsatisfactory n(%)**				
Age(in years)#	32 (28—36)	34 (29—39)	0.96 (0.93—0.993	*0.017	1 (0.95—1.06)	0.995
Number of children#	2 (1—3)	2 (2—3)	0.88 (0.73—1.04)	0.135	1.01 (0.77—1.34)	0.938
**Gender**						
Male	57(32.8)	117(67.2)	0.19 (0.12—0.29)	* < 0.001	0.20 (0.12—0.33)	** < 0.001
Female	156(72.2)	60(27.8)	Ref		Ref	
**Residence**						
Urban	191(52.9)	170(47.1)	0.36 (0.15—0.86)	*0.021	0.53 (0.19—1.45)	0.217
Rural	22(75.9)	7(24.1)	Ref		Ref	
**Languages**						
Urdu	143(59.3)	98(40.7)	1.02 (0.38—2.78)	0.967	1.07 (0.34—3.33)	0.903
Punjabi	42(54.5)	35(45.5)	0.84 (0.29—2.43)	0.748	1.05 (0.32—3.42)	0.940
Sindhi	13(40.6)	19(59.4)	0.48 (0.15—1.58)	0.228	0.78 (0.20—3.03)	0.718
Pashto	5(21.7)	18(78.3)	0.19 (0.05—0.78)	*0.020	0.30 (0.06—1.42)	0.129
Other regional language	10(58.8)	7(41.2)	Ref		Ref	
**Education**						
Illiterate	8(44.4)	10(55.6)	0.48 (0.17—1.38)	0.175	0.22 (0.07—0.76)	*0.017
Primary	17(44.7)	21(55.3)	0.49 (0.22—1.09)	0.081	0.47 (0.18—1.20)	0.115
Secondary	52(45.2)	63(54.8)	0.50 (0.27—0.92)	*0.026	0.52 (0.26—1.04)	0.064
Graduate	93(62)	57(38)	0.99 (0.54—1.78)	0.964	0.81 (0.42—1.57)	0.527
Post-graduate	43(62.3)	26(37.7)	Ref		Ref	
**Family system**						
Joint	81(66.9)	40(33.1)	2.1 (1.34—3.3)	**0.001	1.63 (0.96—2.75)	0.071
Nuclear	132(49.1)	137(50.9)	Ref		Ref	

*CI* Confidence interval, *OR* Odd ratio, *Ref* Reference category, #Variables are expressed as median with inter-quartile range

^*^Significant at *p* < 0.05

^**^Significant at *p* < 0.01

## Discussion

Our study was designed to assess the knowledge, attitude, and practices of parents regarding the red flags of developmental milestones.

Out of the four domains of children’s development, parents were most knowledgeable about language milestones with over 25 percent of respondents showing adequate knowledge. These findings suggest that in our population, perhaps parents monitor their children’s linguistic development more vigilantly than other domains of development. A similar study done in the USA also showed that knowledge about language development was stronger than other domains although they only assessed the knowledge of mothers, and while our study depicts the knowledge of both parents, the results, however, are similar [15]. The fact that linguistic development especially concerns parents is reiterated by results obtained by inquiring about parental practices. 91 percent of parents admitted that they spent time with their children with an explicit intent of improving and aiding their children’s language and social development. This encouraging statistic highlights an opportunity for healthcare professionals to avail; with such an overwhelming majority (91 percent) of parents actively demonstrating vigilance and great concern about their children’s development, adequate measures, if taken, to educate parents on red flag signs will profoundly help facilitate early diagnosis. However, an alarmingly high number of respondents did not have correct knowledge regarding the age at which a child responds to his/her own name and this we assume may be because in our set-up, parents may be on the look-out for the first words but would probably not notice the age at which a child starts responding to his/her own name and thus, would not overlook this important milestone even though a delay in this milestone may also be a red flag for the autism spectrum.

Parents generally tend to place more emphasis on certain motor milestones as compared to other milestones. For instance, in the parent's view, being able to walk is an immensely important milestone in the child's development and thus this milestone rarely goes unnoticed. This had led to the authors expecting adequate parental knowledge regarding the age at which a child walks independently but unfortunately, only 37 percent of respondents were correctly able to answer the pertinent question. Similarly, the authors had expected fair to adequate parental knowledge regarding other well-known motor milestones, e.g., sitting without support., but surprisingly, an overwhelming 81 percent of respondents answered the pertinent question incorrectly. Knowledge regarding motor milestones was overall inadequate with gross motor being lower than fine motor. Parents seemed more confident about knowing when a child should be able to reach for an object, which is a component of fine motor than knowing at what age the child should be able to walk in a straight line, a component of gross motor which was answered incorrectly by 88.2 percent of the parents. Even though a study done in Saudi Arabia showed that parents in that region focused more on the motor milestone than the other domain and their results showed that 50% of the parents had adequate knowledge about motor milestones [2]. The reason for this difference could stem from the fact that the education level of parents in Pakistan varies from the education levels of parents in Saudi Arabia due to differences in literacy rates. It is possible that what parents in the Middle East region of the world consider a developmental delay, parents in Pakistan assume it to be within the range of normal development and hence do not shed light on it. We assume that they may use reasons such as genetics, diet, physical activity, and the environment to be the cause of the late achievement of a milestone and may not get their child treated on time for his/her delay. Hence the parents must be aware of the average age of reaching a developmental milestone to avoid permanent damage to their child’s motor skills which could affect their quality of life as they grow up [16].

Knowledge regarding motor and language milestones did give better results than social which shows that primary concerns of parents are based on their movements and speech rather than their ability to socialize [7]. A similar study done in Saudi Arabia proves that although overall knowledge regarding developmental milestones was poor, knowledge specifically regarding motor milestones was indeed better than the other domains, shedding light on the fact that delays in language and motor are easily noticed by the parents while social and cognitive go unnoticed [2].

From all questions regarding social milestones, the correct responses only came from 9.7% of the parents showing a huge gap in the knowledge of social milestones. The question that received the most incorrect responses was the age at which a child should be able to participate in group play. Only, 14 percent of our respondents answered correctly. In one of the studies done in Aseer, Saudi Arabia shows that only 12.5%, slightly higher than our population [17]. This shows that overall knowledge regarding social milestones is poor regardless of the region of the world. Poor parental knowledge regarding social development should be of great concern to healthcare workers and the Pakistani community. In the case of a child being autistic, parents with inadequate knowledge will most likely overlook his/her abstention from social engagement which will invariably lead to a delay in diagnosis and intervention [18].

Assessing the socio-demographic characteristics of our respondents led to a rather surprising revelation: Parents living in joint families were less knowledgeable about language milestones than those living in nuclear families. This was statistically significant. Ideally the scenario should have been that people belonging to joint family system should be more knowledgeable regarding developmental signs because of more kids around them but in this study we did not found it to be true. We speculate that this is because parents in nuclear families, being the only available caregivers for their children, take a keener interest in acquiring knowledge, develop a more cautious attitude and exercise more vigilance than parents in joint families. Moreover, there may be more children in joint family systems leading to overlooking of certain milestones of individuals. Parents have a tendency of evaluating their child’s development by comparing their activities and behavioral patterns with those of other children of similar age [10]. Some parents who participated in a similar study conducted in Iran were of the opinion that delays in speech and language milestones are not necessarily a cause for concern, as this is a “hereditary aspect” and children will reach these milestones eventually [19]. The authors had anticipated new parents to be less knowledgeable than parents who had had children previously, but contrary to this expectation, the results showed that number of children does not significantly impact parental knowledge (OR = 0.87, 95% CI 0.63–1.18, *p* = 0.359).

The correct attitude and practices of parents may be protective factors in a child’s development. The findings of our study showed a significant association between the female gender and positive attitude. This is perhaps because even in our study, the primary caretakers were mothers. Therefore, mothers also spent the maximum amount of time spent with children and thus there are increased chances of them looking up developmental milestones. A study reported that mothers’ knowledge mainly came from their own mother’s experiences or family sources, therefore it can be assumed that women are more likely to learn from observing children around them, contacting others for information, and are more likely to have a better understanding of a child’s growth compared to fathers [1]. 88 percent of parents reported taking their children to the pediatrician at least once a year; this is a trend that should be looked upon favorably and attempts should be made to incorporate parental education in these visits e.g. distribution of printed brochures containing information about developmental milestones. We anticipate such measures to positively impact parental knowledge.

Our results also show a significant association between the female gender and education when it comes to practices and attitude. Educated parents are more likely to use resources available to them for maximum benefit for themselves and for their children. It is possible that parents with lesser education or lesser resources available may compare their child’s growth with other children and use that as a means of assessment of their child’s growth [19]. Educated parents are more likely to be empowered with an understanding of when and how to interact with children to improve their cognitive, social, and language development. They are also more likely to do their own research to know whom to approach in case help is required. Our findings are consistent with other studies which report the association of better maternal education with better caregiving practices [20]. Furthermore, a recent study involving LMICs demonstrated that both early child economic well-being, as well as the education status of the primary caregiver, predict receptive vocabularies at the age of 5 years [21]. Educated mothers were found to have a positive attitude towards development which means they did believe in getting a clinical assessment done if their child showed any unusual signs or how they think that their pediatrician provides them with sufficient information regarding milestones. We believe that this is a very important factor that helps in the early diagnosis of a developmental delay compared to a mother with a lower level of education who realizes the unusual signs in her child's development but does not know that this is a delay that requires intervention and has the negative attitude that the pediatrician has not provided her sufficient information. Mothers, being mostly the primary caregivers are more observant about milestones and invest more time and energy with the intent of bettering their child’s development [22]. Mothers' knowledge usually comes from previous experience with children or learning about development from children around them however, our study shows that it is not just the fathers who need improvement in learning about a child’s developmental milestones but mothers also need to improve their knowledge to fulfill the criteria of having adequate knowledge. An important factor that this study highlights is that parental education did not play a significant role in having adequate knowledge about developmental milestones. Previous studies done in north India state a similar fact about mothers’ education [10]. Many studies conducted in the past mainly focused on mother’s knowledge and attitude regarding a child’s development [23] hence we included fathers in this study as well and found that there is no significant difference between adequate and inadequate knowledge of both mothers and fathers indicating that both genders need equal awareness regarding development. The difference in the relation between education and knowledge versus the association of education with attitude and practices may be that while the parents may be educated, they may not necessarily have the correct knowledge regarding developmental milestones [24].

Perhaps the reason behind this is that when we asked parents about their sources of information, many answered that they either learn from the internet or the child’s grandparents. A few parents also mentioned that pediatricians have been a great help in helping them learn about their children’s developing milestones. Regardless, the overall knowledge remains inadequate in all domains hence we must have educational programs for parents where they learn the signs of developmental delay and act as early as possible in case of a developmental delay, as a study done previously in the USA has shown a positive response in parent’s awareness regarding milestone, we hope for a similar response from our population [25, 26].

### Limitations

The main limitation that we acquired in our study was a small sample size and since we were only able to collect the data from one hospital in one city, there is a chance that data from different cities would have produced different results. Another limitation that we came across was the language barrier. We could only take data from those who were fluent in English or Urdu, eliminating those who spoke any other language. Thus, to have a bigger picture, studies must be conducted in every city and include people who speak different languages to get better results. Moreover, we did not come across a validated tool for assessment that could have been adapted for resource-poor countries such as Pakistan, so a self-designed questionnaire was used. Since our questionnaire was self-designed, we only focused on four domains including gross motor, fine motor, social and language and could not directly include other domains such as cognition.

## Conclusion

Although knowledge was overall inadequate in all four domains, our study indicated relatively better knowledge of gross and fine motor milestones as compared to language and social milestones. With regards to attitude and practices, the female gender had a positive association, which highlights that although knowledge levels do not differ between genders, mothers have the right approach in dealing with indicators of developmental delay. Our study highlights the need to devise ways to educate parents on this subject to enable them to vigilantly monitor their child’s developmental status and any associated abnormalities and ultimately facilitate the right course of action.

### Supplementary Information

**Additional file 1.**

## Data Availability

The datasets used and/or analyzed during the current study are available from the corresponding author upon reasonable request.

## Abbreviations

^a^CI: Confidence interval
OR: Odd ratio
Ref: Reference category
#: Variables are expressed as median with inter-quartile range
