# Supplementary material for: Knowledge, attitude, and practices of parents regarding the red flags of developmental milestones in children aged 0–5 years in Karachi, Pakistan: a cross-sectional study
Source: BMC Pediatr. 2024 Feb 14;24:120. doi: 10.1186/s12887-024-04574-9 (PMC10865706; doi:10.1186/s12887-024-04574-9)
Supplement: Supplementary file 1 — Additional file 1. [file 12887_2024_4574_MOESM1_ESM.pdf]

Supplementary material title: Questionnaire- “Knowledge, Attitude, and Practices of parents regarding the red flags of developmental milestones in children aged 0-5 years in Karachi, Pakistan: A cross-sectional study”

Liaquat National Hospital and Medical College

Informed Consent

You are invited to participate in an interview for a research study titled “Knowledge, attitude and practices of parents regarding red flags of developmental milestones in children.” This is a study being conducted by Dr. Raman Kumar, Paediatric Neurologist at the department of Paediatric Medicine, and MBBS students at Liaquat National Hospital and Medical College.

Your Participation in this study is entirely voluntary and consensual. You reserve the right to refuse to answer any particular question for any reason. Furthermore, you may choose to withdraw from the interview at any point at your discretion. Your responses will be stored in a protected online format to ensure confidentiality. You may choose to remain anonymous.

In case your child is found to have a developmental delay during the course of this interview, further evaluation will be provided free of cost.

I have read and understood the Research consent form and its content. I consent to being interviewed for this study

Name مtí ______________________________ **Signature** ɑz¡sد ____________________

Personal Information

(Please circle the applicable option in each row)

Gender	Male/Female/Other
Age in years	
Residence	Urban/Rural
Current Residence	
First Language	Urdu/Punjabi/Pashto/Sindhi/Balochi/Other: ______________
Religion	Islam/Hinduism/Christianity/Sikhism/Parsi/Other: __________
Education	Illiterate/Primary/Secondary/Matriculation/Intermediate /Graduate/Post Graduate
Occupation	
Marital Status	Married/Separated/Divorced/Widowed
Cousin Marriage	Yes/No
Number of Children	
Ages of Children (Oldest to Youngest)	
Family System	Joint/Separate
Average Monthly Household Income	Less than Rs. 20,000<br>Rs. 20,000-Rs.50,000<br>Rs.50,000-Rs.100,000<br>Rs. 100,000-Rs. 200,000<br>Rs. 200,000-Rs.500,000<br>More than Rs. 500,000
Primary caretaker for children	Mother/Father/Grandmother/Grandfather/Aunt/Sibling/ Maid/Other: __________

Knowledge regarding Gross Motor milestones

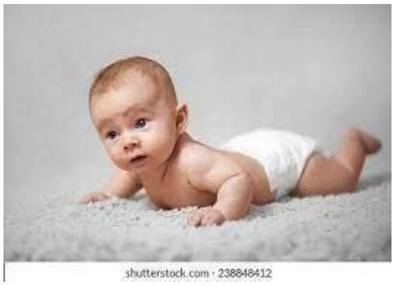 https://www.alamy.com/stock-photo-baby-boy-on-his-stomach-learning-to-lift-head-111659954.html	1.By what age should a child be able to lift his head and chest while lying on stomach?	A. 1 month<br>B. 3 months<br>C. 6 months<br>D. 10 months
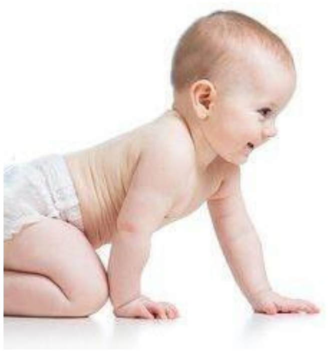<br>https://www.mindmoves.co.za/2018/01/19/will-crawling-style/	2. By what age should a child be able to crawl and sit without support?	A. 2 months<br>B. 4 months<br>C. 6 months<br>D. 10 months
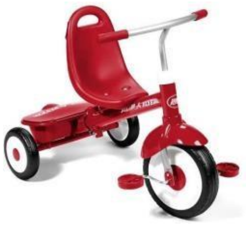<br>https://m.media-amazon.com/images/I/81mxzNPIvVL.jpg	3. By what age should a child be able to pedal a tricycle?	A. 1 year<br>B. 2 years<br>C. 3 years<br>D. 5 years

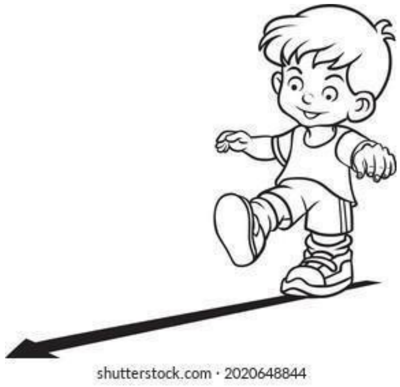<br>https://www.shutterstock.com/tr/image-vector/child-walking-on-straight-line-exercising-2020648844	4. By what age should a child be able to walk along a straight line?	A. 1 year<br>B. 2 years<br>C. 3 years<br>D. 5 years
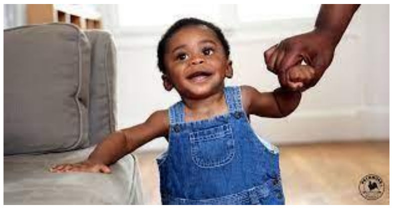<br>https://www.primroseschools.com/blog/5-exercises-to-help-your-baby-walk/	5. By what age should a child be able to walk with support e.g with one hand held or by holding onto furniture?	A. 6 months<br>B. 12 months<br>C. 16 months<br>D. 20 months
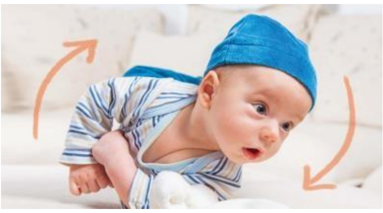<br>https://www.mamanatural.com/when-do-babies-roll-over/	6. By what age should a child be able to roll over in either direction?	A. 2 months<br>B. 4 months<br>C. 6 months<br>D. 10 months
7. By what age should a child be able to walk up and down stairs with one hand held?		A. 12 months<br>B. 2 years<br>C. 2.5 years<br>D. 3 years

<table>
<tr>
<td>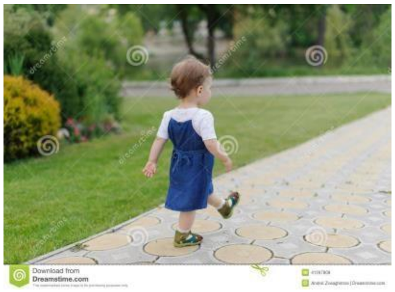
dreamstime.com</td>
<td>8. By what age should a child walk independently?</td>
<td>A. 8 months
B. 12 months
C. 18 months
D. 24 months</td>
</tr>
<tr>
<td colspan="2"><u>Red Flag</u></td>
<td>By what age did your child start walking?

___________months

In case your child wasn’t able to walk independently, by what age would you have been concerned enough to consult a pediatrician?

_____________months</td>
</tr>
<tr>
<td>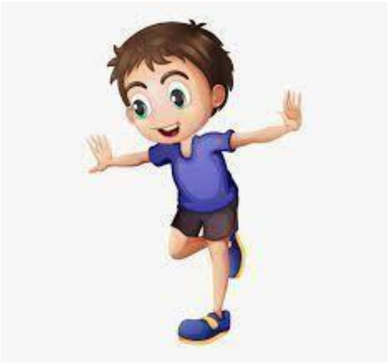
https://www.vhv.rs/viewpic/bTwRiR_stand-on-one-foot-clipart-hd-png-download/</td>
<td>9. By what age should a child be able to stand on one foot for a few seconds?</td>
<td>A. 1 year
B. 2 years
C. 2.5 years
D. 4 years</td>
</tr>
</table>

Knowledge regarding Fine Motor milestones

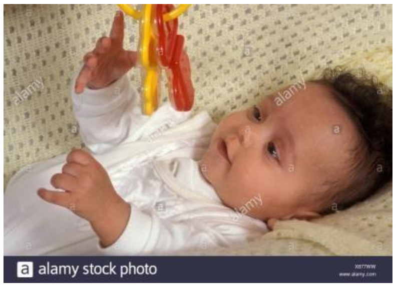<br>alamy.com	1. By what age should a child be able to reach for objects e.g rattle after seeing them?	A. 1 month<br>B. 3 months<br>C. 6 months<br>D. 1 year
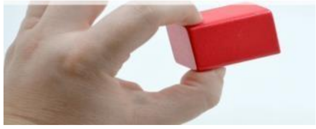<br>https://www.growinghandsonkids.com/what-is-a-pincer-grasp.html	2. By what age should a child be able to do a pincer grasp i.e hold an object with index finger and thumb?	A. 2 months<br>B. 6 months<br>C. 10 months<br>D. 18 months
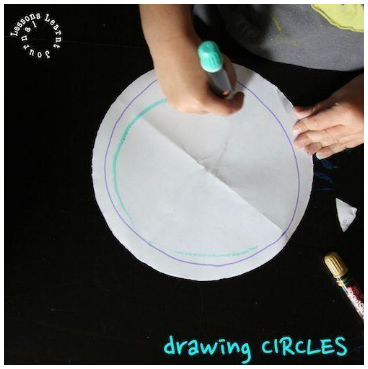<br>http://lessonslearntjournal.com/drawing-circles/	3. By what age should a child be able to copy a circle?	A. 10 months<br>B. 18 months<br>C. 2 years<br>D. 3 years
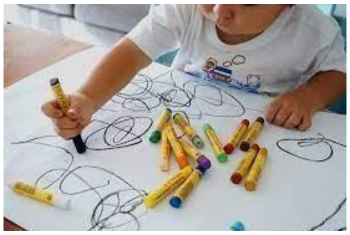<br>https://www.google.com/url?sa=i&url=https%3A%2F%2Fwww.toledolibrary.org%2Fblog%2Fyouth-art-month-it-all-starts-with-a-simple-box-of-crayons&psig=AOvVaw348MjWlASXv6hpM9n3s4Ep&ust=1697463604466000&source=images&cd=vfe&opi=89978449&ved=0CBEQjRxqFwoTCODduOuW-IEDFQAAAAAdAAAAABAE	4. By what age should a child be able to scribble with a pen/pencil?	A. 4 months<br>B. 8 months<br>C. 12 months<br>D. 18 months

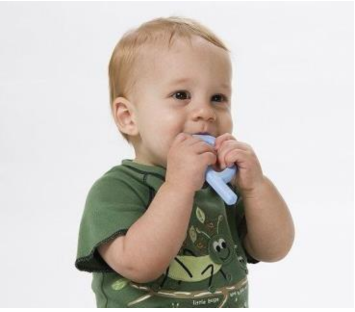<br>https://www.arktherapeutic.com/blog/why-do-babies-put-everything-in-their-mouths/	5. By what age should a child put objects in his/her mouth?	A. 1 month<br>B. 3 months<br>C. 8 months<br>D. 18 months
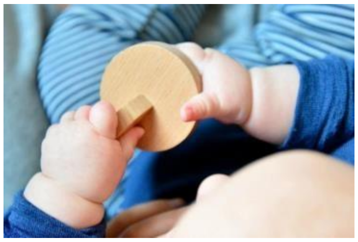<br>https://www.howwemontessori.com/how-we-montessori/2018/02/give-toys-with-purpose-montessori-toys-for-grasping-and-hand-to-hand-transfer.html	6. By what age should a child be able to transfer objects from one hand to the other?	A. 1 month<br>B. 3 months<br>C. 6 months<br>D. 10 months
**<u>Red Flag</u>**		By what age was your child able to transfer objects from one hand to the other?<br>_____________months<br><br>In case your child wasn't able to transfer objects from one hand to the other, by what age would you have been concerned enough to consult a pediatrician?<br>_____________months
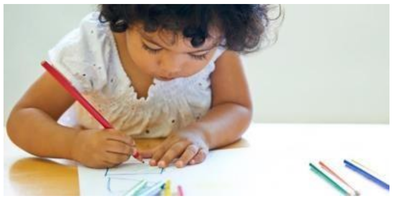<br>https://www.playfulbee.com/blog/2017/11/13/learning-simple-shapes/	7. By what age should a child be able to copy a square or a triangle?	A. 1 year<br>B. 2 years<br>C. 3 years<br>D. 5 years

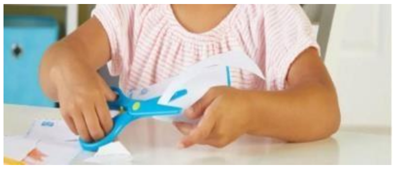<br>https://www.learningresources.co.uk/blog/fine-motor-skills-milestones/	8. By what age should a child be able to use scissors to cut out a figure/picture?	A. 1 year<br>B. 3-4 years<br>C. 4-5 years<br>D. 6 years
9. By what age should a child be able to close a box with a lid?		A. 6 months<br>B. 1 year<br>C. 2 years<br>D. 4 years

Knowledge regarding Social milestones

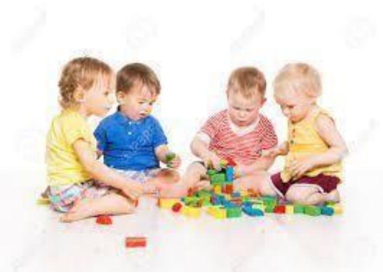<br>https://www.bigstockphoto.com/tr/image-85371929/stock-photo-children-group-playing-toy-blocks-little-kids-early-development-baby-activity-one-year-old-games/	1. By what age should a child be able to participate in group play?	A. 1 year<br>B. 2 years<br>C. 2.5 years<br>D. 5 years
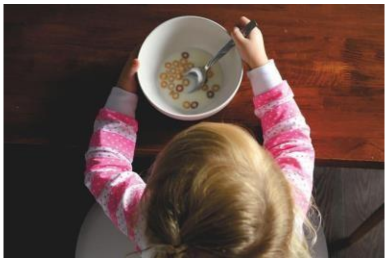<br>https://parenting.firstcry.com/articles/when-your-toddler-picks-up-a-spoon-for-feeding/	2. By what age should a child be able to drink from a cup and use a spoon?	A. 12 months<br>B. 18 months<br>C. 2 years<br>D. 4 years

<table>
<tr><td>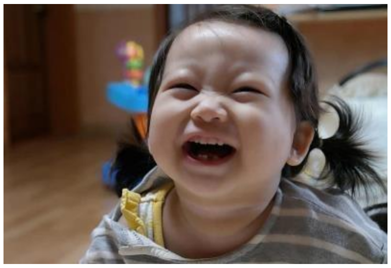
https://earlylearningnation.com/2021/04/crying-is-easy-laughing-is-hard/</td><td>3. By what age should a child be able to smile spontaneously?</td><td>A. 2 months
B. 6 months
C. 8 months
D. 10 months</td></tr>
<tr><td colspan="2"><u>Red Flag</u></td><td>At what age did your child first smile?
__________months

In case your child didn't smile, by what age would you have been concerned enough to consult a pediatrician?
__________months</td></tr>
<tr><td colspan="2">4. By what age does a child develop a fear of strangers?</td><td>A. 3 months
B. 8 months
C. 12 months
D. 18 months</td></tr>
<tr><td>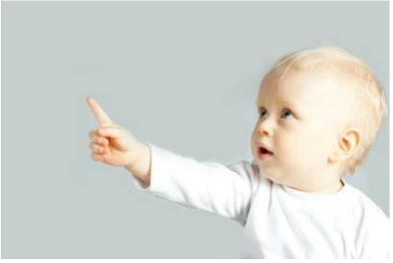
https://www.parentlane.com/toddler/toddler-development/your-child-will-show-you-his-wants-by-pointing-at-the-object</td><td>5. By what age should a child be able to point to a desired object?</td><td>A. 3 months
B. 6 months
C. 12 months
D. 2 years</td></tr>
</table>

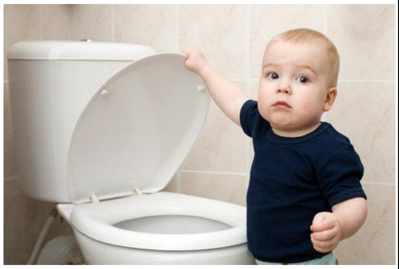<br>https://parentingscience.com/potty-training-age/	6. By what age should a child be toilet trained?	A. 3 years<br>B. 5 years<br>C. 7 years<br>D. 8 years
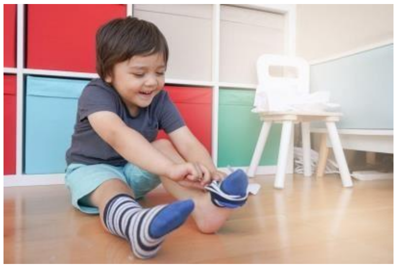<br>https://www.performancehealth.com/articles/how-to-teach-your-child-with-special-needs-to-dress-independently	7. By what age should a child be able to remove garments like shoes and socks?	A. 8 months<br>B. 12 months<br>C. 18 months<br>D. 2 years
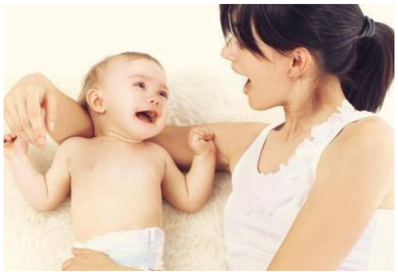<br>https://parenting.firstcry.ae/articles/when-and-how-do-babies-recognize-their-name/	8. By what age does a child normally begin to recognize his/her caregiver?	A. 1 month<br>B. 2 months<br>C. 5 months<br>D. 10 months
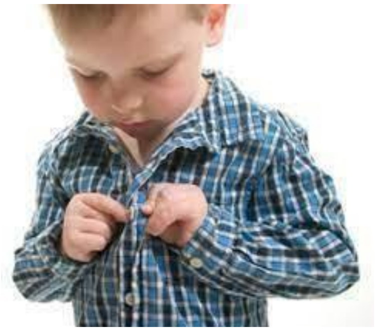<br>https://www.raepica.com/wp-content/uploads/2019/04/child-buttoning-shirt.jpg	9. By what age should a child be able to dress and undress except tying shoe laces?	A. 2 years<br>B. 4 years<br>C. 6 years<br>D. 7 years

Knowledge regarding Language milestones

1. By what age does a child begin to respond to his/her own name?	A. 5 months<br>B. 10 months<br>C. 18 months<br>D. 24 months
2. By what age does a child say his first word?	A. 4 months<br>B. 11 months<br>C. 18 months<br>D. 2 years
3. By what age should a child be able to vocalize when talked to?	A. 1 month<br>B. 3 months<br>C. 6 months<br>D. 12 months
4. By what age should a child have a vocabulary of 50 or more words?	A. 6 months<br>B. 8 months<br>C. 12 months<br>D. 24 months
**Red Flag**	By what age was your child's speech easily understandable?<br>____________years<br><br>In case your child's speech wasn't clearly understandable, by what age would you have been concerned enough to consult a pediatrician?<br>____________years
5. By what age should a child be able to tell which hand is right and which is left?	A. 1 year<br>B. 2 years<br>C. 3 years<br>D. 5 years

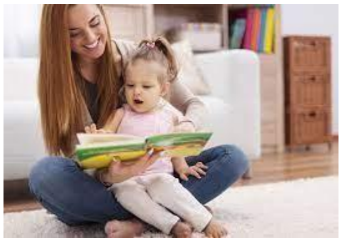<br>https://blog.earlymoments.com/how-important-is-it-to-read-to-kids/	6. By what age does a child enjoy being read to?	A. 12 months<br>B. 18 months<br>C. 3 years<br>D. 5 years
7. By what age should a child be able to give his/her full name and age?		A. 2 years<br>B. 3 years<br>C. 5 years
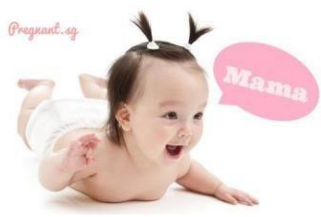<br>pregnant.sg	8. By what age should a child be able to call his mother and father “mama” and “dada” respectively?	A. 2 months<br>B. 6 months<br>C. 10 months<br>D. 18 months
9. By what age should a child be able to respond to simple instructions like “sit down” or “bring it here”?		A. 8 months<br>B. 12 months<br>C. 18 months<br>D. 3 years

Questions: Parental Attitude and Practices

1. Have you ever looked up/sought information for childrens’ developmental milestones yourself?

- Yes
- No

If Yes, what is your primary source for gathering information regarding childrens’ developmental milestones?

- Internet
- Parents (Grandparents of child)
- Relatives
- Pediatrician

- Other: _____________

2. Pediatricians have provided me with satisfactory and sufficient information regarding childrens' developmental milestones and their red flags.

Strongly Disagree	Disagree	Neutral	Agree	Strongly Agree
1	2	3	4	5

3. How frequently do you visit a pediatrician yearly?

______________________times yearly

4. How much time do you spend with your child daily?

- Less than 1 hour
- 1 to 2 hours
- 2 to 4 hours
- 4 to 8 hours
- More than 8 hours.

5. Spending time interacting with children is important for their language and social development.

Strongly Disagree	Disagree	Neutral	Agree	Strongly Agree
1	2	3	4	5

6. A certain developmental delay may run in families.

Strongly Disagree	Disagree	Neutral	Agree	Strongly Agree
1	2	3	4	5

7. Any developmental delays will be a major cause of concern for me.

Strongly Disagree	Disagree	Neutral	Agree	Strongly Agree
1	2	3	4	5

8. Delays in motor development are a strong indication of physical disability.

Strongly Disagree	Disagree	Neutral	Agree	Strongly Agree
1	2	3	4	5

9. Delays in social and verbal development can lead to the child becoming deaf and/or mute.

Strongly Disagree	Disagree	Neutral	Agree	Strongly Agree
1	2	3	4	5

10. Who should one consult if their child has a developmental delay?

A. General Paediatrician
B. Pediatric Neurologist
C. Developmental Pediatrician
D. Family Physician
